# Supplementary material for: Asymmetric dysregulation of glutamate dynamics across the synaptic cleft in a mouse model of Alzheimer’s disease
Source: Acta Neuropathol Commun. 2023 Feb 14;11:27. doi: 10.1186/s40478-023-01524-x (PMC9926626; doi:10.1186/s40478-023-01524-x)

Supplementary Figure 1

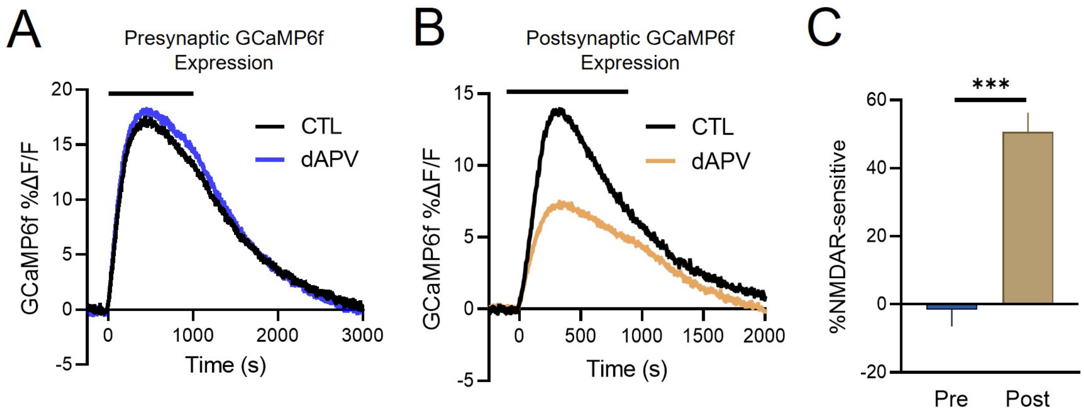

Supplementary Figure 2

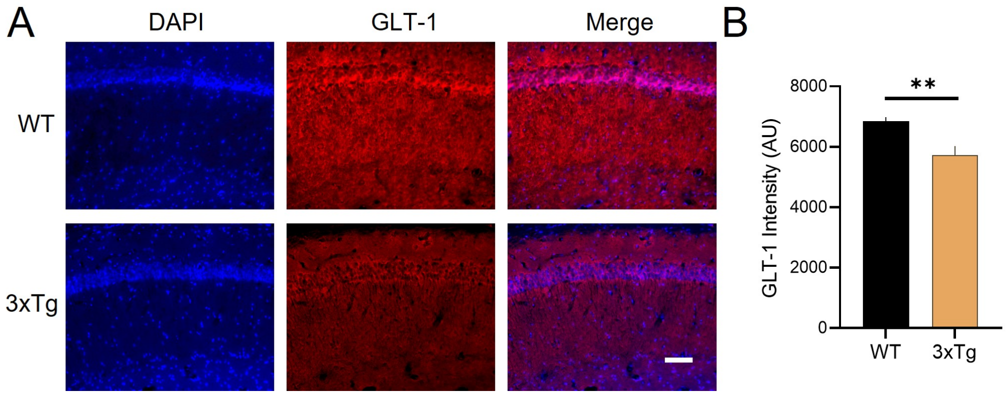

Supplementary Figure 3

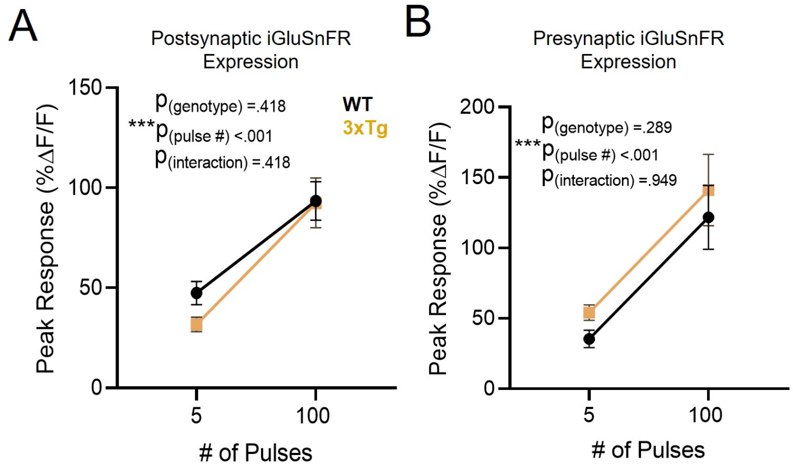

Supplementary Figure 4

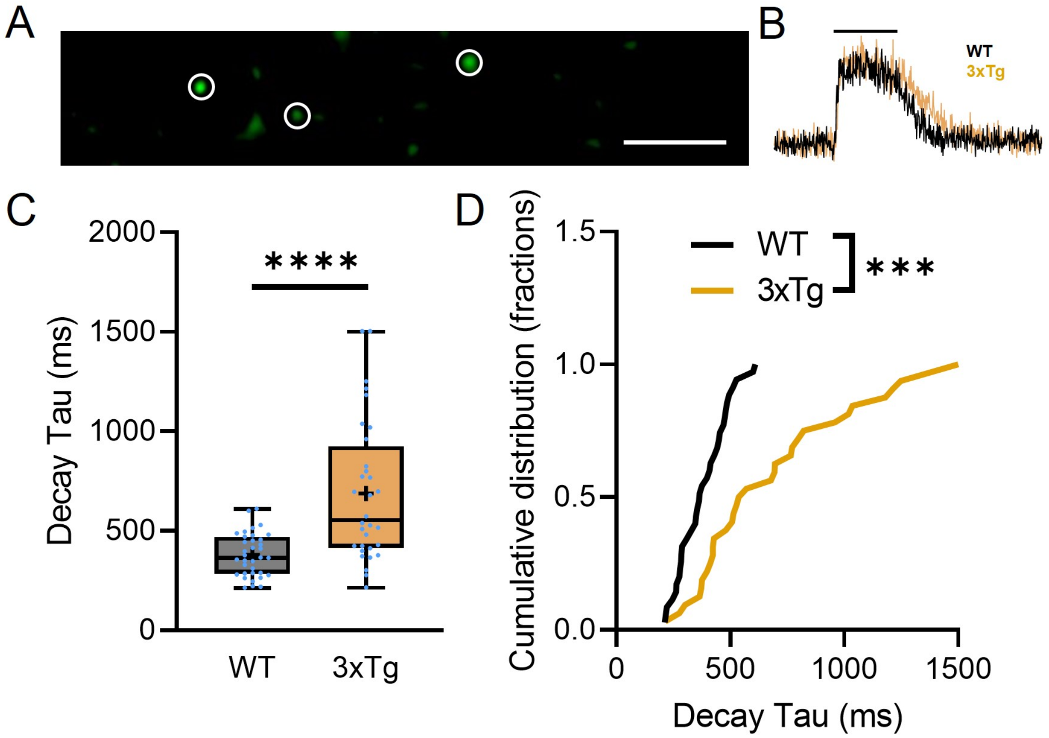

Supplementary Figure 5

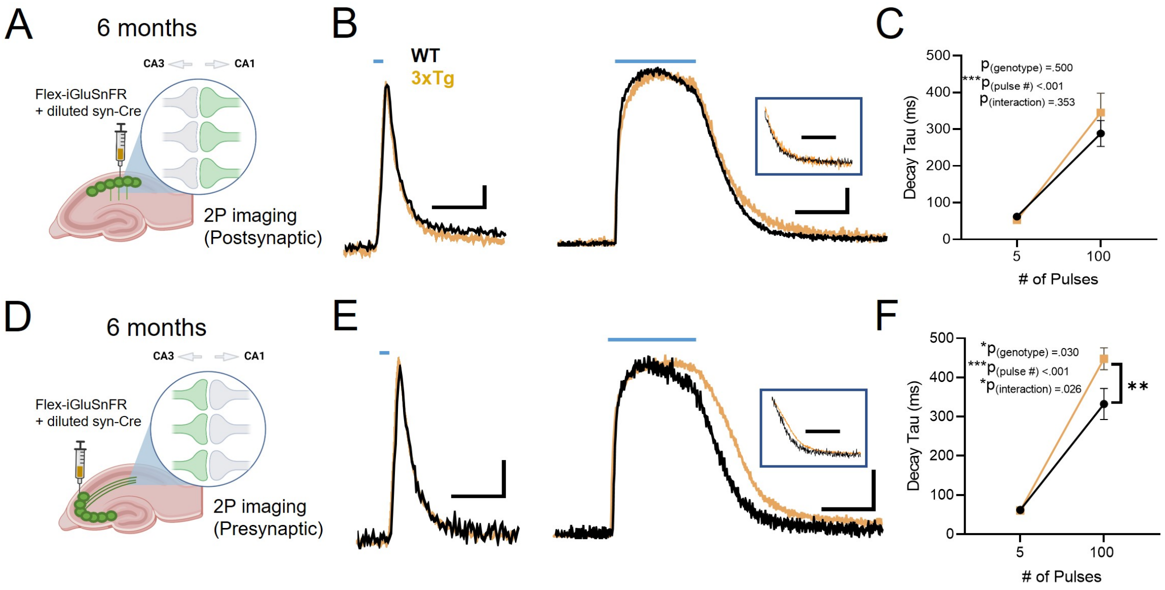

Supplementary Figure 6

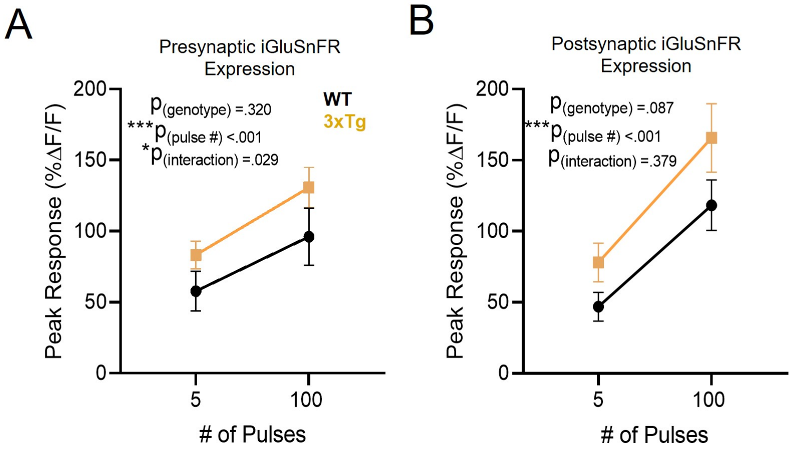

Supplementary Figure 7

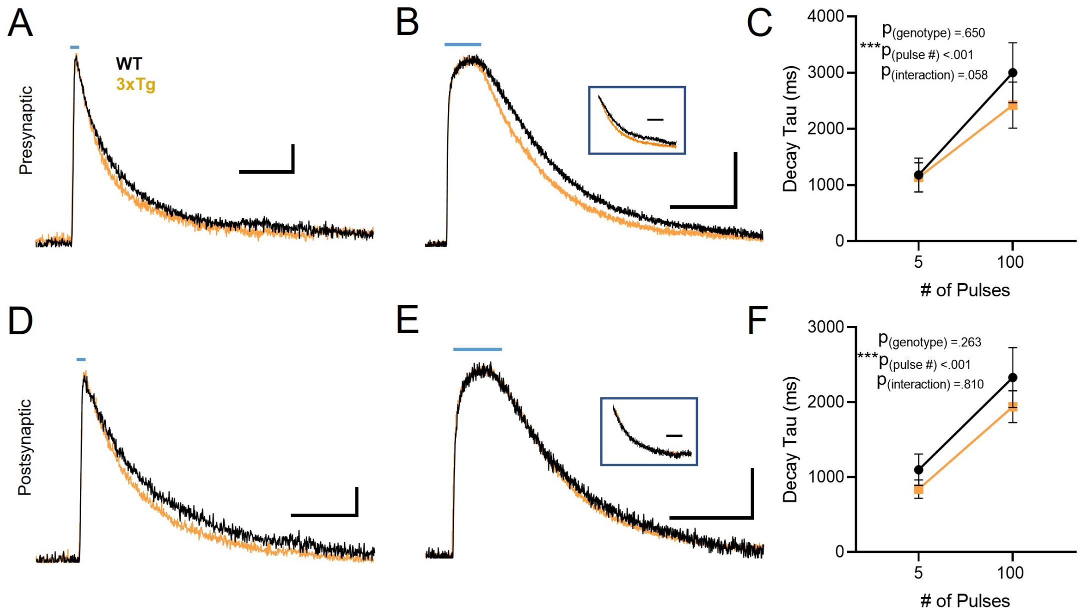

Supplementary Figure 8

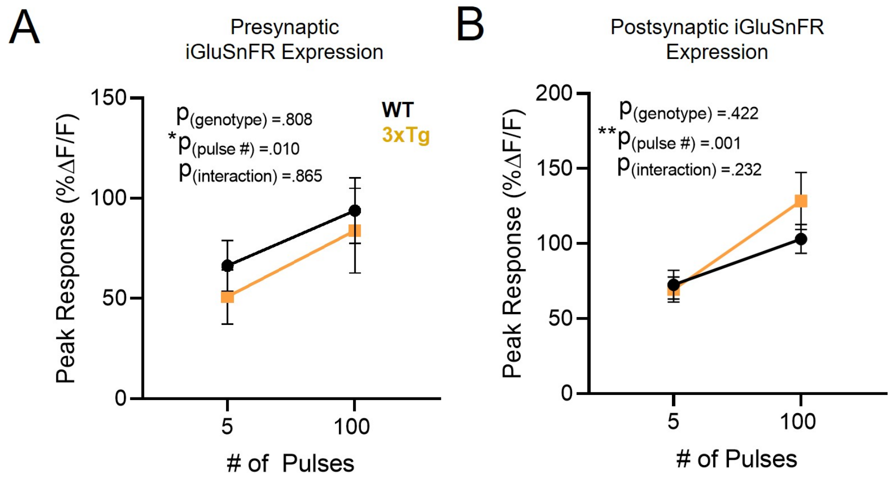

Supplementary Figure 9

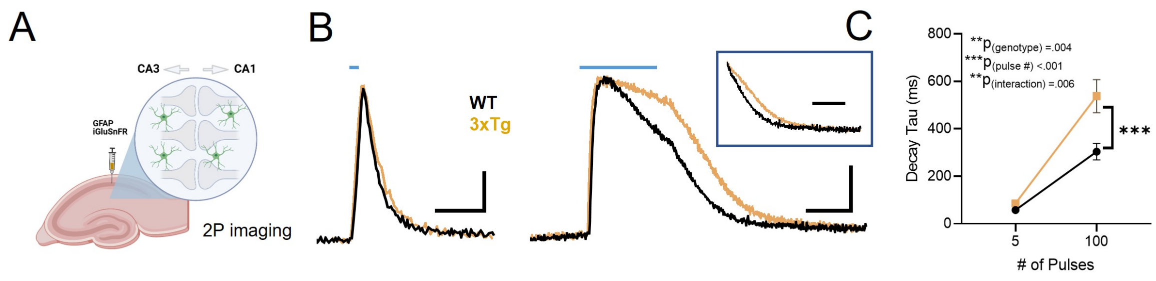

Supplement: Supplementary file 1 — Additional file 1. Figure 1. NMDA receptor blockade reduces postsynaptic but not presynaptic calcium responses to high-frequency stimulation. (A) Presynaptic GCaMP6f response to high-frequency stimulation (HFS) before (black) and after (blue) bath application of d-APV (50 μM). B Postsynaptic GCaMP6f response to HFS before (black) and after (orange) d-APV. C postsynaptic GCaMP responses are more sensitive to NMDAR blockade than presynaptic GCaMP responses, with a 50% reduction observed in the postsynaptic GCaMP response. Error bars represent s.e.m. *** p < 0.001. Figure 2: GLT-1 expression is significantly reduced in 3xTg hippocampus. WT and 3xTg mice were perfused at 6 months of age. GLT-1 intensity was quantified in stratum radiatum. All immunostaining was performed at the same time and imaging parameters (LED intensity, exposure times) remained consistent for both genotypes. WT n = 12, 3xTg n = 10. Scale bar in A: 50 μm. Error bars represent s.e.m. ** p < 0.01. Figure 3. Peak iGluSnFR responses do not differ between WT and 3xTg mice. A Postsynaptic iGluSnFR response peaks in WT (black) and 3xTg (orange) mice. B Presynaptic iGluSnFR response peaks in WT (black) and 3xTg (orange) mice. Figure 4. iGluSnFR dynamics at individual iGluSnFR-positive puncta are slower to decay in 3xTg mice. (A) Representative image showing presynaptic iGluSnFR expression. ROIs are drawn around individual iGluSnFR puncta representing putative single synapses. B Representative iGluSnFR responses to electrical stimulation (100 pulses, 100 Hz, indicated by the black line above the traces). C Box-and-whisker plots of putative single synapse decay tau values following stimulation. Individual ROI responses are shown as dots within the plot. (D) Cumulative distribution plot of the decay tau values at the quantified ROIs. Scale bar in A: 10 µm. *** p < 0.001, **** p < 0.0001. Figure 5. Presynaptic glutamate clearance impairment and spared postsynaptic clearance in the 3xTg hippocampus replicated a [file 40478_2023_1524_MOESM1_ESM.pdf]
